# Validating therapeutic COPE cards in sexual assault survivors: converging psychophysiological, behavioral, and neural evidence within the SEE FAR CBT framework for PTSD

**DOI:** 10.1192/j.eurpsy.2026.11870

**Published:** 2026-07-31

**Authors:** S. Raz, M. Lahad

**Affiliations:** 1 The Center for Psychobiological Research, Yezreel Valley College, Yezreel Valley; 2 Psychology, Tel-Hai Collge, Upper Galilee; 3 Community Stress Prevention Center, Kiryat Shmona, Israel

## Abstract

**Introduction:**

SEE FAR CBT (Somatic Experience–Fantastic Reality–Cognitive Behavioral Therapy) is an integrative, trauma-focused treatment for PTSD and anxiety disorders. It has been applied in clinical practice for over two decades, with consistent positive feedback from patients and therapists regarding its effectiveness and acceptability. Its *Fantastic Reality* component refers to the capacity of individuals under extreme stress to enter a protected imaginative space, where traumatic experiences can be externalized and playfully transformed, enabling survivors to engage creatively with difficult material while maintaining emotional safety. This process is activated in therapy through COPE cards—associative artistic drawings—designed to evoke emotional states and facilitate therapeutic engagement. Although used clinically, empirical validation of COPE cards remains limited.

**Objectives:**

To evaluate the efficacy of COPE cards in eliciting differential psychophysiological, behavioral, and neural markers of emotional arousal in women who had experienced sexual assault and consequently presented with symptoms of PTSD.

**Methods:**

Two complementary studies were conducted.

**Psychophysiological study:** Participants completed baseline recordings of heart rate (HR), blood pressure (BP), and heart rate variability (HRV), then viewed negatively-valenced (arousing) cards followed by positively-valenced (safe) cards, with measures taken after each phase (Fig 1).

**Neurocognitive study:** Participants viewed negative, ambivalent, and positive cards while providing ratings, mismatch detection, and reaction times (RT), alongside EEG recordings of brain event-related potentials (ERP: P1, P2, P3, LPP).

**Results:**

In study 1, relative to baseline, exposure to negative (stressful/arousing) cards increased HR and BP and reduced HRV, while subsequent exposure to positive (safe) cards produced the opposite pattern, reflecting differential ANS reactivity (Fig 2). In study 2, ratings showed a valence gradient (positive highest, ambivalent intermediate, negative lowest). Ambivalent cards elicited the most mismatches. RTs differed across valence conditions, with ambivalent stimuli eliciting the slowest responses, followed by negative and then positive stimuli. ERP analyses revealed robust modulation by emotional condition, with negative cards eliciting enhanced P1, P2, P3, and LPP amplitudes, consistent with heightened neural sensitivity to negative cues (Fig 3).

**Image 1:**
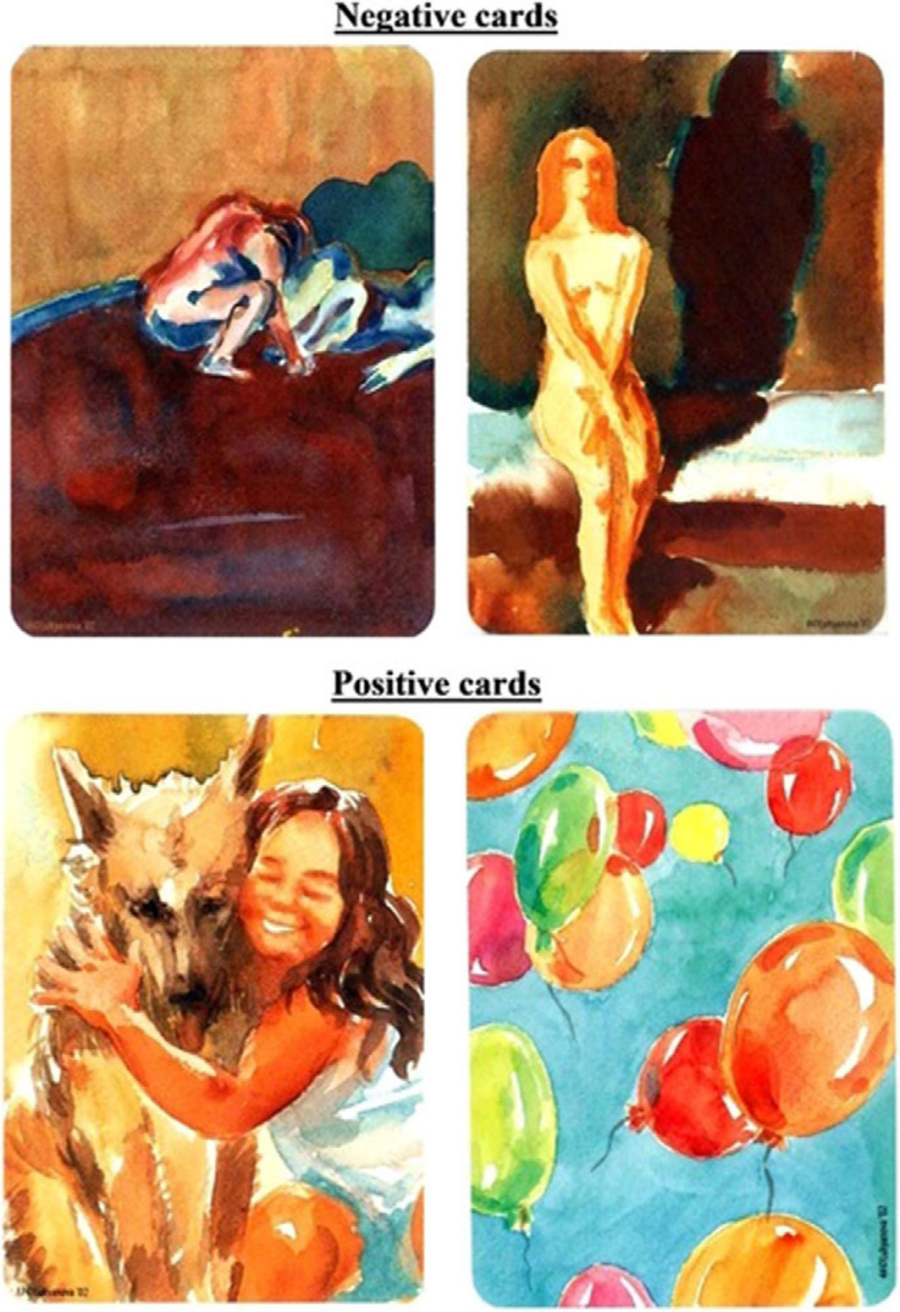

**Image 2:**
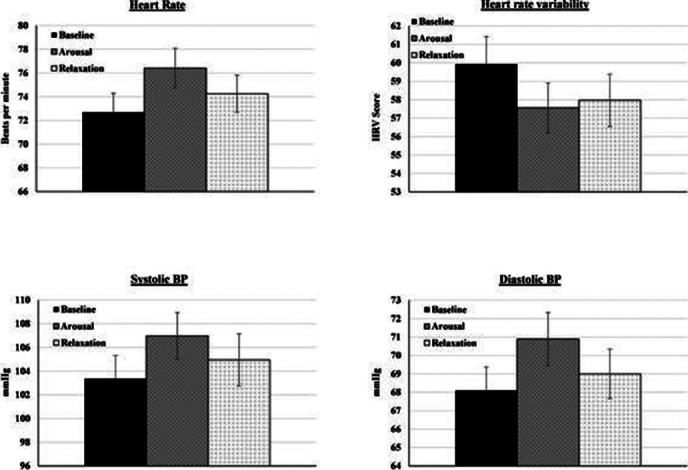
Image 2: Long description.

**Image 3:**
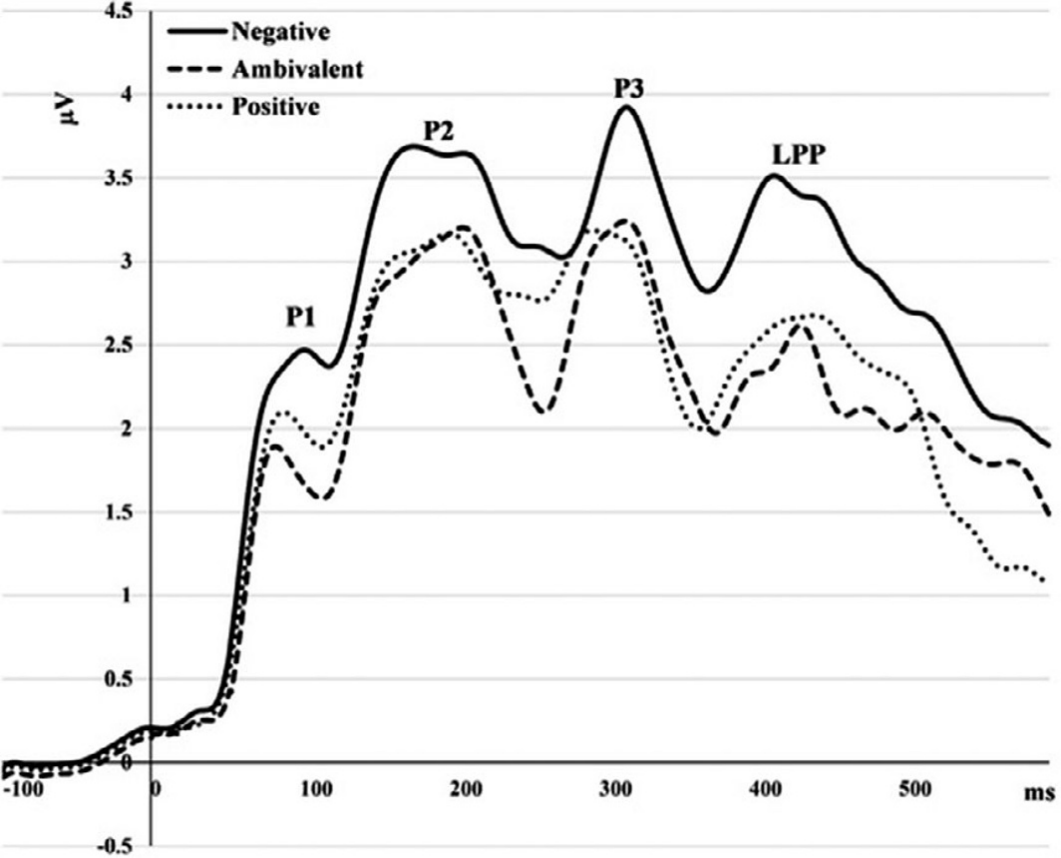
Image 3: Long description.

**Conclusions:**

Across physiological, behavioral, and neural domains, COPE cards reliably elicited distinct arousal-affective states, providing empirical support for their use as therapeutic tools. Within the SEE FAR CBT framework, they offer a playful yet safe means of influencing emotional processing and facilitating symbolic transformation of trauma-related material.

**Disclosure of Interest:**

None Declared

## Long descriptions

### Image 2: Long description

Four bar charts arranged in a two-by-two grid. Each chart includes error bars and a legend identifying three states: Baseline (solid black), Arousal (gray texture), and Relaxation (dotted pattern).

* Top-left chart: Heart Rate. The y-axis is Beats per minute ranging from 66 to 80. Baseline is approximately 72.5. Arousal increases to approximately 76.5. Relaxation decreases to approximately 74.

* Top-right chart: Heart rate variability. The y-axis is H R V Score ranging from 53 to 62. Baseline is highest at approximately 60. Arousal is lowest at approximately 57.5. Relaxation is approximately 58.

* Bottom-left chart: Systolic B P. The y-axis is m m H g ranging from 96 to 110. Baseline is approximately 103.5. Arousal increases to approximately 107. Relaxation decreases to approximately 105.

* Bottom-right chart: Diastolic B P. The y-axis is m m H g ranging from 64 to 73. Baseline is approximately 68. Arousal increases to approximately 71. Relaxation decreases to approximately 69.

Navigate back to Image 2:.

### Image 3: Long description

The x-axis represents time in m s from minus 100 to 600. The y-axis represents amplitude in mu V from minus 0.5 to 4.5. Three lines are plotted. A solid line for Negative, a dashed line for Ambivalent, and a dotted line for Positive. All three lines show a sharp increase starting at 50 m s.

* P 1 peak occurs around 100 m s with the Negative line highest at 2.5 mu V, followed by Positive and Ambivalent.

* P 2 peak occurs around 180 m s where the Negative line reaches 3.7 mu V while Ambivalent and Positive lines cluster near 3.2 mu V.

* P 3 peak is the highest inflection point at 300 m s with the Negative line peaking at 4.0 mu V and the other two lines peaking at 3.2 mu V.

* L P P peak occurs around 420 m s with the Negative line at 3.5 mu V and the others near 2.7 mu V.

From 450 m s to 600 m s, all three waveforms show a gradual decline, with the Negative line maintaining the highest amplitude throughout the entire duration.

Navigate back to Image 3:.

